# Manuskript zweier Linzer Apotheker aus dem Jahre 1529 mit Anweisungen zur Verhütung und Behandlung der Pest

**DOI:** 10.1007/s10354-025-01092-w

**Published:** 2025-07-09

**Authors:** Heinz Flamm

**Affiliations:** 1 Martinstraße 7, 13400 Klosterneuburg, Österreich; 2 Medizinische Universität Wien, Wien, Österreich

**Keywords:** Rezepte für Schutz vor Pest, Pflanzen gegen Pest, Aderlass, Verhalten gegen Pest, Recipes for protection against plague, Plants against plague, Bloodletting, Behaving in plague times

## Abstract

Wenige Jahre nach Druck (1521) der ersten Pestordnung in den Habsburgischen Ländern, aber ganz unabhängig davon, berichten zwei Apotheker in Linz an der Donau, Oberösterreich, in einer 32-seitigen Handschrift über verschiedene Krankheiten, dabei aber ganz ausführlich über Vorkehrungen, die für den einzelnen Menschen zur Verhütung einer Infektion mit der Pest zu unternehmen sind. Es wird dafür die Einnahme von speziellen Latwergen und von Zubereitungen verschiedener Pflanzen, insbesondere aus deren Wurzeln, empfohlen. Wenn jedoch die ersten Zeichen der Erkrankung bereits aufgetreten sind, ist ein an bestimmten Körperstellen gesetzter Aderlass sinnvoller. Die Angaben wie lange ein solcher noch erfolgreich sein kann, sind an mehreren Stellen des Manuskripts unterschiedlich mit zwei bis 24 h angegeben.

Das umfängliche Manuskript dient offensichtlich, anders als die Pestordnungen, nicht der Anleitung für Obrigkeiten zur Erhaltung der Volksgesundheit, sondern die Autoren haben wohl für Laien, Ärzte und Bader geschrieben.

## Abstract (en)

A few years after the print of the first plague instruction in the Habsburg countries (1521) and certainly independent thereof two pharmacists in Linz an der Donau, Upper Austria, in their manuscript on 32 pages reported about various illnesses and especially about measures for the prevention of plague in the individuals. They recommended the intake of special electuaria and preparations of different plants and their root-stocks. If the first signs of the illness already have appeared venesections are recommended on certain spots of the body. In different parts of the manuscript the indication of the tolerated time between the onset of the sign and the intervention varies between two and 24 h.

The manuscript obviously, in contrast to official plague instructions, was not intended to give guidance for official measures in favour of the public health, but to give guidance to individuals, physicians and treaters.

Im 16. Jahrhundert begann die verwaltungsmäßige Bekämpfung der Pest in den Habsburgischen Erblanden durch die Erstellung von Pestordnungen. Als erster Fürst ließ Erzherzog Ferdinand^1^ „Ein nutzliche ordnũg vnd regimẽt wider die Pestilentz durch Doctor Hansen Saltzman võ Steir“, seinem Leibarzt, erstellen, die am 15. September 1521 „zw nutz der Ersamen Lanndschafft“ für Graz und ganz „Innerösterreich“ (Steiermark, Kärnten und Krain) erlassen wurde. Im Jahr 1534 folgten die „Oberösterreichischen Länder“^2^ mit der in Sterzing erlassenen „Ordnung in den sterbenden Leůffn der Pestilentz“. Wien und die „Niederösterreichischen Länder“ (Österreich ob und unter der Enns) erhielten 1540 ihre Anordnung „Wie mañ sich zů zeiten der Pestilentz fürsehen vnd erhalten mög“. Das damals von Österreich noch unabhängige Fürstliche Erzstift Salzburg betonte 1547 in seiner „Ordnung zu abstellung der vnsaubrigkait, hie in der Stat“ die Wichtigkeit der Sauberhaltung der Orte, damit nicht „sonnderlichen in den Sterbennden Leuffen“ [*Pest*] „pöse Krannckhaiten entspringen“.

Die in den Pest- oder Infektionsordnungen verlangten Maßnahmen gegen die Seuche betrafen vorwiegend Vorkehrungen gegen den Ausbruch und die Ausbreitung der Seuche. An erster Stelle stand die Vermeidung der Kontakte, insbesondere mit Erkrankten und die in der frühen Neuzeit stark vernachlässigte Sauberkeit nicht nur der Menschen selbst sondern auch ihrer Umwelt. (Genaueres über die Pestordnungen des 16. Jahrhunderts für die gesamten Habsburgischen Länder siehe bei Flamm [1]).

Die Pestordnungen haben sich an die offiziellen Institutionen gewandt, die sich für die Gesundheit der Bevölkerung einsetzen müssten. Es erschien mir daher als angenehmer Zufall, dass ich durch die freundlichen Bemühungen von Herrn Franz Siegle, Antiquar in Tübingen, eine Handschrift aus dem Jahre 1529 erwerben konnte, die vom Maister Paulsen, Appotekher zű Lintz, und vom Appoteckher Dominicűs zű Lintz verfasst war (Abb. 1). Sie trägt den Titel „Regement vnd Ártzneӳ in dem Englisch Schwais – 1529 – Vnd am lesten steet für die Breӳn • Ich noch zü lest für die Pestelentz • Vnd noch am blat ain Cöstlich bewart stückh vom Hanfsamen alten vnd jungen vnd vergifften sachen“ (*Breӳn, Prein, (Häutige) Bräune* *=* *Diphtherie*). Die Handschrift besteht aus 38 durch einen Faden gebundenen Blättern von ca. 15 × 20 cm und zwar aus einem Titelblatt, 30 Blättern mit beidseitigem Text, einem einseitig beschriebenen Blatt sowie sechs freien Blättern.

**Abb. 1:**
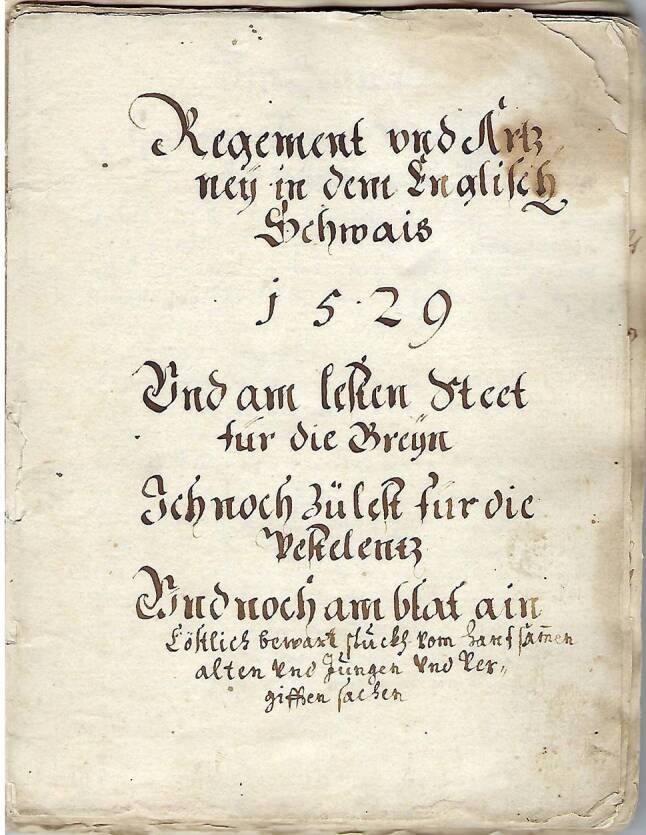
Titelblatt der Handschrift

Beide Apotheker sind weder in Linz an der Donau noch, nach schriftlicher Auskunft, in Linz am Rhein nachweisbar. Es ist aber als sicher anzunehmen, dass beide Herren im österreichischen Linz an der Donau tätig waren. Bereits am Anfang des Manuskripts [p. 4] steht, dass die verabreichte Artzneӳ bei „kindsblatern“ [*Feuchtblattern*], den „bösen Laὑff“ [*Pest*] und dem „Englischen Schwaiß“ im Welschlandt [*Welschtirol* *=* *Trentino*] geholfen hat. Es wird auch über eine versuchte Behandlung von „Sandt vnd grieß“ in „der blatter“ [*Harnsteine*] beim „ro. kn. [*röm. königl.*] Postmaister zὑ Wien“ berichtet [p. 14]. Des Weiteren erfährt man bei der Besprechung von Schutzanhängern, dass der bereits in obiger Einleitung als Initiator der ersten Pestordnung in österreichischen Landen genannte König Ferdinand sich über solche Schutzmittel informiert hat und sie offenbar auch selbst benützt hat [p. 58]. Aus der Erwähnung Ferdinands als König ergibt sich die Zeit zwischen 1526 und 1561.

Die Autorenschaft der vielen einzelnen Abschnitte ist nicht angegeben. Eine Auskunft gibt das erste Blatt in der Überschrift „Im Schwaiß • Regiment von Maister Paulsen. Appotekher Zű Lintz“ mit Anweisungen für das Verhalten von Kranken, die an Englischem Schweiß litten (über Englischen Schweiß siehe Flamm [2]).

Der folgende namentlich von Apoteckher Dominicűs verfasste Text behandelt neben der Pest noch verschiedene Krankheiten wie u. a. die „Prein“ [*Diphtherie*], „die Würmb im Leib der Kinder“, „Schwindtel“, das „Blűetten [*Bluten*] der Nasen“, die „Podagra“ [*Gicht*], das „Zendwee“ [*Zahnweh*], „Wantzen“, den „krawit baűmb“ [*Wacholderbaum*], die „Rebarbara wűrzen“ [*Rhabarberwurzel*] und die „Rüehr“ [*Ruhr*]. Weiters findet man in dem umfangreichen Manuskript keine Hinweise auf die Autorenschaft der einzelnen Abschnitte.

Hier will ich insbesondere die in den Abschnitten „In der Pestilentz – Artzeneӳ vom Dominicűs Apoteckher zű Lintz, In Ihr Jahr geschicht“ (p. 3, Abb. 2), „Fűr die Pestilentz“ [p. 9], „Regiment für die Pestilenz“ [p. 10] und „Vnderricht der Läß vnd Artzeneÿ contra Peste“ [p. 35] sowie in einzelnen Abhandlungen des Manuskripts gebrachten Angaben vereinen. Diese enthalten zum Teil einander widersprechende Angaben, was wohl auf die Zusammenarbeit zweier (oder mehrerer) Autoren hinweist.

**Abb. 2:**
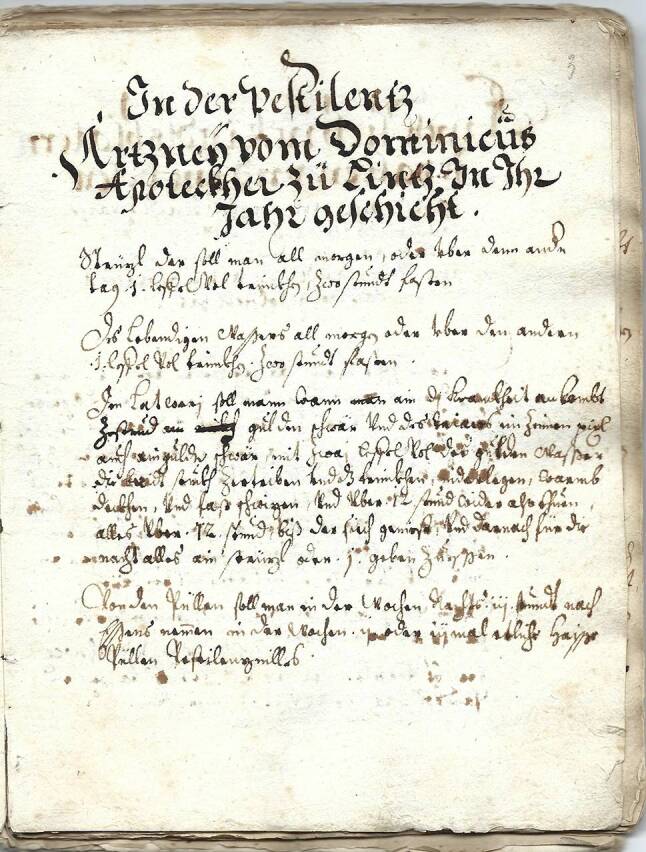
Eine Abschnittsüberschrift

## Vorbeugung der Erkrankung

Der „Vnderricht der Läß^3^ vnd Artzeney contra Peste“ beginnt mit einer allgemeinen Bemerkung, „wie sich der Mensch in den gemainen Laüf^4^ halten solt, mit Arzeney vnd mit Weißheit“.

Ganz wichtig für jedem Einzelnen war die Verhütung der Pest-Erkrankung bereits zu der Zeit „dieweil der sterb wart“, also bereits vor Auftreten der ersten Krankheitsfälle. Aus den sich sehr oft wiederholenden Beschreibungen ist allerdings nicht immer klar, ob der Text die Vorbeugung, die Behandlung oder beide betrifft.

**Abb. 3:**
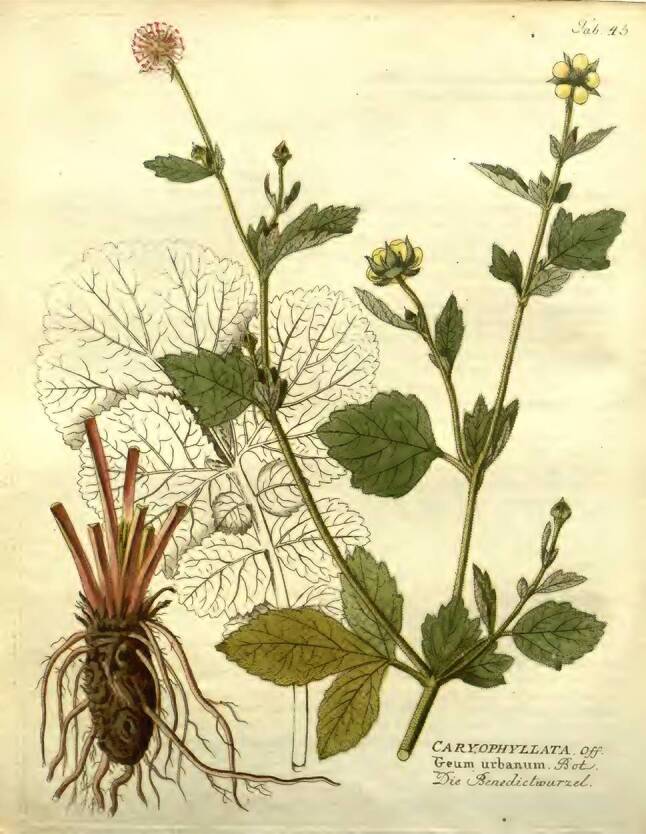
Benediktenkraut. Vietz FB [3]. 1800.1. Band, Tab. 43. (Die Abb. 3, 4, 5, 6 und 7 wurden freundlicherweise von der Österreichischen Nationalbibliothek, Wien, zur Verfügung gestellt)

Neben der Vermeidung des Kontakts mit Kranken werden verschiedene Gegenmaßnahmen empfohlen. Eine einfache ist, jede Nacht ein Pulver aus der Wurzel von „Pabstkraut genant Corda Benedicta“ [*Benediktenkraut* *=* *Nelkenwurz, Geum urbanum*] in Essig einzunehmen (Abb. 3).

Auch die Wurzel des Teufelsabbisses [*Succisa pratensis*] ist zur Vorkehrung „guet in disen leüffen“. Dazu wird die Wurzel, in einigen Rezepturen die ganze Pflanze, im Mörser zu einem ganz feinen Pulver zerrieben und dieses durch ein Tuch gesiebt und in ein Glas mit bestem Wein gegeben. Von dem nach etwa einem halben Tag klar gewordenen Wein soll man dann täglich morgens nüchtern „einen gueten trunckh machen, vngefehrlich [*ungefähr*] ain claines becherlain volß“.

Aber auch „alle Morgen nüchtern einen Trunkh Hanifmilch [*Hanf-Milch*] gethan, hilft davor, sonderlich ainigen leuthen“, die Pest zu bekommen. Hanf wurde in der Zeit zur Behandlung und Vorbeugung bei verschiedenen Krankheiten verwendet. So empfiehtl der eingangs erwähnte Appotekher Maister Paulsen, man soll den an „Englischem Schweiß“ Erkrankten zur Behandlung „auch haniff milch zetrinkhen geben“

Man kann aber auch Kranabet pör [*Kranabitbeeren, Wacholderbeeren*] vorbeugend gegen Pest verwenden, z. B. indem man sie ein- bis zweimal in der Woche morgens in Essig zerstoßen in einer sauren Speise isst. Wacholderbeeren werden auch als Latwerge (*latwȇrge, latwȇrje (mhd.)* *=* *breiige Arznei, Mus*) verwendet. Wer morgens nüchtern die Latwerge zu sich nimmt, ist 24 h lang sicher vor der Krankheit und sogar auch wenn er Kranke besucht. Es wird auch empfohlen, täglich die Latwerge und ein Stück Zitwer-Wurzel [*Artemisia cina, eine Beifußart*] zu sich zu nehmen.

**Abb. 4:**
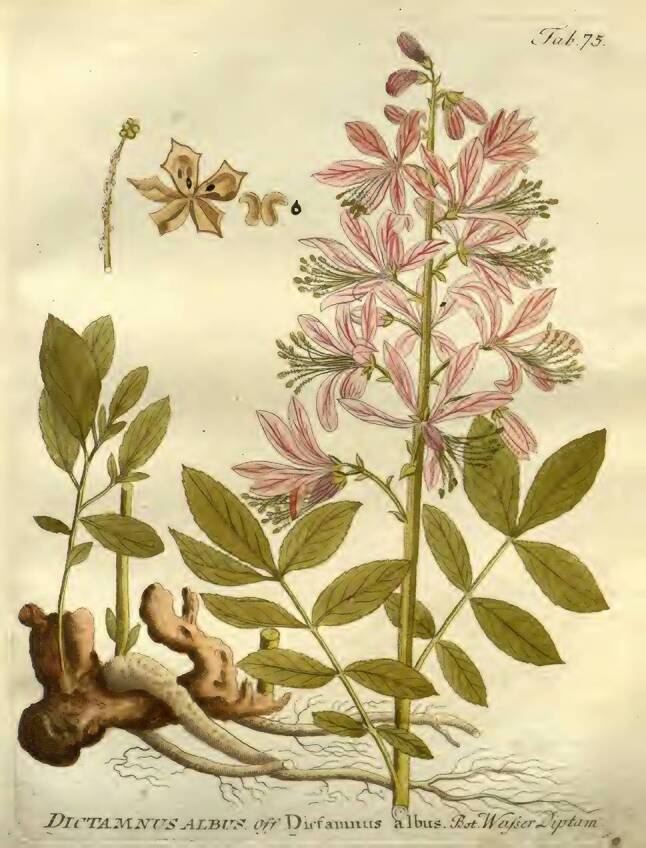
Diptam. Vietz FB [3]. 1800. 1 Band, Tab. 75

Die Herstellung einer besonderen Pest-Latwerge ist wesentlich aufwendiger: In ein Ei macht man ein kleines Löchlein, durch welches das Eiklar entfernt wird. Zum verbliebenen Dotter gibt man ungestoßenen Safran und verschließt das Loch mit einer anderen Eischale. Dann legt man das Ei zu einem Feuer und lässt es langsam braten bis die Schale überall ganz braun geworden ist. Nach Zerstoßen des Eies in einem Mörser gibt man dieselbe Menge „weißen seniff“ [*Senf, Sinapis alba*] dazu und nach Verarbeitung zu Pulver weiters noch je ein Lot [*1 Lot* *=* *16 *^*2*^*/*_*3*_ *g*] von gestossener Wurzel des „weissen Tüptans“ [*Weißer Diptam, Dictamnus albus*] und der Tormantilla [*Fingerkraut, Blutwurz, Tormentilla erecta*] sowie Kraut von Cronaigl [*Bunte Kronwicke, Coronilla varia*] (Abb. 4, 5 und 6). Alles wird zu besonders feinem Pulver zerrieben. Danach wird die Masse eventuell je nach Rezept mit verschiedenen Zugaben versehen und mindestens zwei weitere Stunden im Mörser zerrieben bis eine Latwerge entsteht, die zäh am Pistill hängen bleibt. Diese Latwerge hält sich in einer Büchse bis zu dreißig Jahre „guet vnd crefftig“. Ob man diese Latwerge auch zur Verhütung der Pest oder erst nach deren Beginn geben soll, ist nicht ersichtlich.

**Abb. 5:**
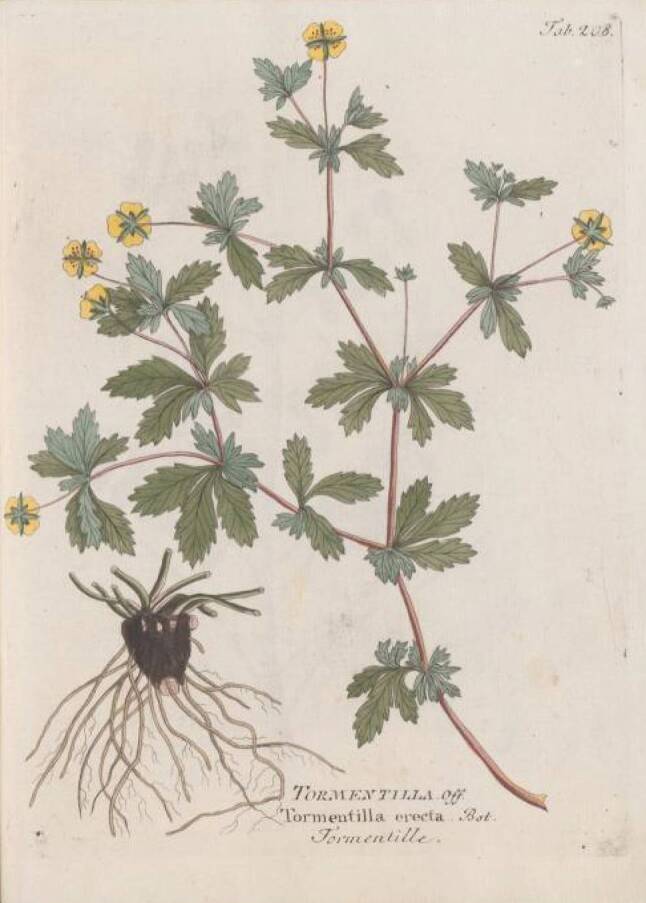
Tormentilla. Vietz FB [3]. 1800. 2. Band, Tab. 218

**Abb. 6:**
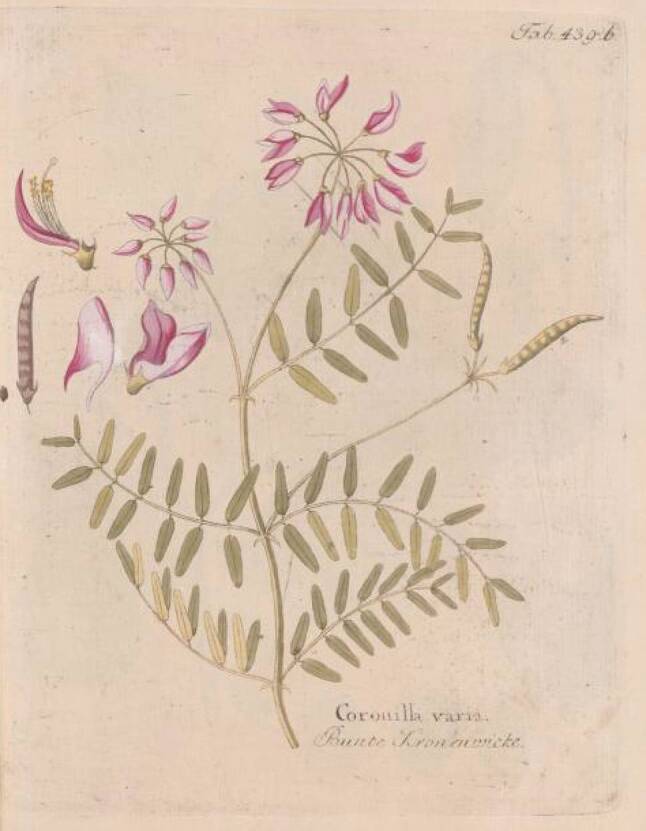
Kronwicke. Vietz FB [3]. 1817. 5. Band, Tab. 439b

Manche Menschen trugen zur Verhütung von Krankheiten verschiedene Amulette an einer Schnur oder Kette um den Hals. Soweit diese Hohlkörper waren – seien es kostbare Geschmeide oder nur einfache Nussschalen – wurden sie mit magischen Abwehrmitteln gefüllt, die zum Teil Düfte ausströmten, weswegen sie auch Bisamäpfel genannt wurden. In der Handschrift wurde empfohlen, den ganzen Tag einen Anhänger mit Teufelsabbiss „am blossen laib an dem herzen“ zu tragen. Dafür wurde ein wenig [*von dem oben beschriebenen Wein oder vom Wurzelpulver?*] in ein Wachstuch und darüber in Atlasgewebe und Samt eingebunden, „damit das gifft gas nicht heraus mag vnd wol verwart sey“, denn „ain gifft das andere [*Gift*] nicht leiden mag“ lässt kein Gift zum Herzen. „Das hat sich [*sic*] könig Ferdinand, sambt anderm abgeschribenem, getraulich gebraucht“.

Zusätzlich oder auch unabhängig von solchen Vorkehrungen empfiehlt es sich, vor dem Schlafengehen in einer Kammer mit versperrten Fenstern und Türen Kranabitbeer-Latwerge und Wermutkraut [*Artemisia absinthium*] in eine Pfanne zu verbrennen und den Rauch durch Mund und Nase einzuatmen. Am Morgen soll man dann nicht lang nüchtern bleiben, denn dies wäre schädlich.

Ein anderes Rezept für „Ain Raüch“ sagt: „nimb Lorber, Cronibeten [*Wacholderbeeren*] vnd Wermuth clain geschnitten auf ain gluot, rauch in allen Zimmern offt damit, vnd das die Zimmer vermacht saind, damit der Raüch darin blaib“.

## Am Beginn der Krankheit

„So den menschen die saich [*Seuche*] ankhumbt“, wenn also die Seuche beim Einzelnen beginnt, so gibt man die Latwerge zerrieben und verdünnt zu trinken, und zwar alten Menschen im Gewicht eines Dukaten [*3,49* *g*], jungen Menschen und Frauen im Gewicht eines Rheinischen Guldens [*= Gewicht von drei Viertel eines Dukatens*]. Dies muss spätestens in der elften Stunde nach Krankheitsbeginn geschehen, denn danach geht das Gift zum Herzen und es würden die Latwerge und auch das Schwitzen nicht mehr helfen. Trotzdem muss man es aber auch noch später versuchen.

**Abb. 7:**
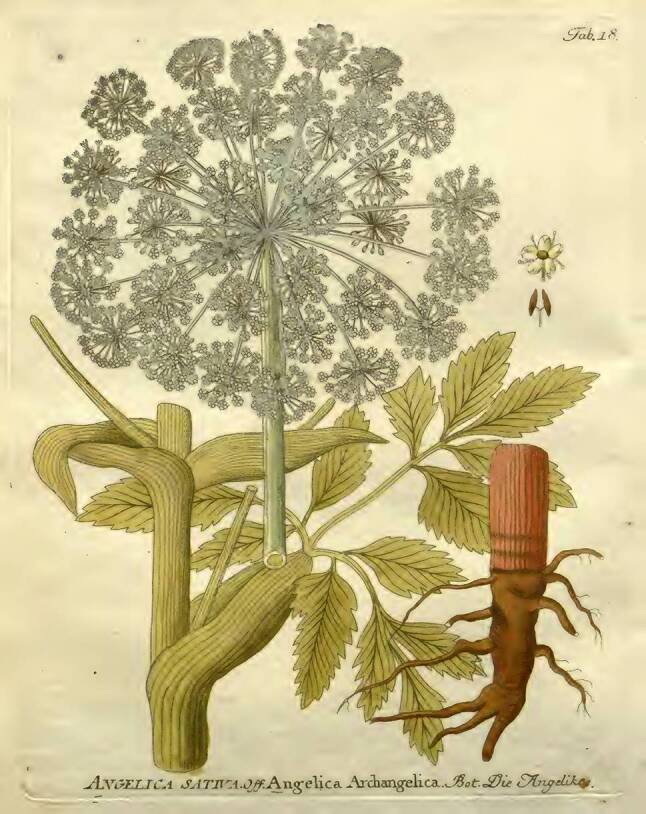
Engelwurtz. Vietz FB [3]. 1800. 1. Band, Tab. 18

Man kann aber auch in Wasser stark zerriebenes Gerstenkorn und Dukatenkraut [*Habichtskraut, Hieracium*] zu trinken geben. Es können auch über Tag Weißer Diptam, Angelica [*Engelwurz, Angelica archangelica*] (Abb. 7) und Weinkraut [*Weinraute, Ruta graveolens*] in Essig eingenommen werden.

Wenn die Pest mit Kälte beginnt, so gibt man einen löffelvoll von der in Weißwein zerriebenen Latwerge zu trinken. Fängt die Krankheit aber mit Hitze an, so nimmt man „blaues Vairlwasser [*Veilchenwasser*] oder Wegward wasser“ [*Gemeine Wegwarte, Wilde Zichorie, Cichorium intybus*]. Man kann auch in Essigwasser oder in frischem „Prunnwasser“ [*Brunnenwasser*] zerriebene Latwerge verabfolgen.

Den Kranken legt man in ein Bett „vnd dekht Ihn auf das allerwermist zue, daß er schwiz, 3, 4 oder 5 stund oder noch lenger, ie mehr ie besser, dan [*denn*] das Gifft der Pestilenz muss sich im schwizen verzehren vnd hinweckh gehen, dann [*wenn*] so eins also lang schwizt, so ist er genossen [*genesen*]. Wolt oder nicht aber der siech [*Sieche*] nit schwizen, so laß von stund 4 Zieglstain warmen, vnd schlag umb ieden ein Naß tuech, leg ihm zween zu den füßen vnd zween zwischen die Pain oder an iede seithen ainen, damit er schwiz, anders genest er nicht“.

„Wan der krankh schwizt, soll man sich hüethen vor seinem adem, vnd dem dampff so von Ihm gehet, damit niemand vergifft werd, man sol auch das Peth vndt Pethgewandt [*Bettzeug*] schön waschen vnd lang erlüfften lassen.“

Wenn man einen Kranken besuchen will, soll man zuvor morgens nüchtern die Latwerge essen, denn das hilft sicher 24 h lang vor einer Ansteckung.

Der Kranke selbst soll acht Tage lang kein Fleisch essen und keinen Wein trinken, „wolte er aber des weins nit gerathen, der ihm doch fast schadt ist [*schadet*], so misch ihm den mit wasser“.

Will der Mensch sicher sein, dass er nicht an der Pest stirbt, so nehme er Solomey^5^, Bösualda, Holunderblüten und weißen Imber (*(mhd.) Ingwer*) und zerstoße sie zu einem Teig und trinke dies in gutem Wein.

## Der Aderlass

Neben oder anstelle der „medikamentösen“ Therapie wird auch der Aderlass angewendet. Die Angaben darüber finden sich an verschiedenen Stellen des Manuskripts und sind widersprüchlich.

Wenn sich bei einem Menschen Zeichen der Pest zeigen, so soll er zur Ader gelassen werden, und zwar in einer Angabe spätestens nach zwei Stunden, in einer anderen innerhalb von 12 h, denn danach „ist das Siechthumb volkommen vnd hilfft dan kain arznay nicht mehr“ und der Mensch muss sicherlich sterben. An anderer Stelle wird angegeben, dass man 24 h nach Auftreten der ersten Zeichen beim Menschen nicht mehr zur Ader lassen sondern purgieren soll. Es ist darauf zu achten, dass niemand schläft, bevor er zur Ader gelassen wurde oder eine Latwerge eingenommen hat.

Nun sollt man wissen, „alles Gifft kúmbt von dem Luft [*mask.!*] so er vergifft ist“. Die Luft vergiftet den Menschen, zerstört ihn und bringt den Tod. Das Gift der Luft geht zuerst in die Hand und dann zum Herzen. Dieses wird „tödtlich“ und sendet das vergiftete Blut zu den Achseln. Wenn es dort nicht hinauskann, geht es zur Leber und danach ins Hirn. So werden dem Menschen drei „Haubt glider ganz zerstört vnd sein Natur [*wird*] krankh vnd bringt dem menschen den Todt“.

Wenn also „am menschen ain zaichen [*der Krankheit*] vnder den Ichsten [*Achseln*]“ auftritt, „so ist das Herz krankh in den Todt“. Wollte man dem Herzen zu Hilfe kommen, so lasst man auf der Hand des gleichseitigen Arms zur Ader, „die da haist die herz adern“. Machte man dies am anderen Arm, brächte dies zweifachen Schaden: gutes Blut würde „außgegossen“ und bei einer abermaligen Erkrankung würde an der [*dann?*] gesunden [*derzeit kranken?*] Stelle vergiftetes Blut „außgegossen“. „So wird dann das blut ganz zerstört zu baiden saithen“, wodurch das Herz stirbt. Demnach soll man immer nur auf dem Arm zur Ader lassen, neben dem das Zeichen aufgetreten ist.

„Erhebt sich aber ain Zaichen bei dem gemächt [*männl. Genitale*] zú nechst bei der Haimblichkait der scham, so sollt Ihr wissen, dass die Leber krank und vergiftet ist. Dann sollt Ihr auf demselben Fuß lassen, und zwar auf der Ader, die zwischen großer und der nächsten Zehe geht, und nicht auf dem Arm, denn das Gift würde zum Herzen oder zum Hirn gezogen und brächte den sicheren Tod.“

„Erhebt sich dann ein Zaichen hinden von der scham (*schame (mhd.)* *=* *Scheu, Rücksicht, Genitale*) und erscheint an den diech [*Schenkel*], so solt Ihr auf demselbigen fὑeß lassen“, und zwar auf der Ader zwischen den kleinen Zehen. Wenn „es auch sticht in saiten“, so lasst „auf der Baslica und der Latain Bathica und auch auf der Ader, die da heißt Salucela und zwischen dem mittleren und dem langen Finger verläuft“.

„Erhebt sich aber ein Zeichen unter den Ohren oder unter dem Kinn, so sollt Ihr auf der gleichseitigen Hand lassen, insbesondere auf der Ader, die da Zevalica heißt und über der medianen Ader auf der Ader zwischen Daumen und Zeigefinger liegt“.

Der Kranke darf nur kurz schlafen, denn kommt er über zwölf Stunden, so hilft das Lassen nicht mehr. „Wer vergifft ist mit dem siechthumb der blatern“ [*hier: Pestwunden*] und/oder „driesen“ [*Drüsen, hier angeschwollene Lymphknoten*] soll z. B. Senfsirup, Holunderblüten und Kräuter-Essig einzeln oder in Mischungen auflegen.

Im Manuskript findet man mitten zwischen anderen Abschnitten auch eine Kurzanleitung für die Auswahl der jeweiligen Stellen zum Aderlass [p. 22]:

„Nemblich so ainem ain zaichen auffart, soll alweg auf derselben saithen gelassen werden, da das zaichen ist.

Also wär es under den ïachsen [*Achseln*], so lass die Media an derselben saithen mitn auf dem armb.

Ists beim gemächten, so lass die roß ader.

Ists auf den lendten, so laß bei der klain zehen.

Ists am knie, so laß die haubt ader beim daumen.

Ists beim orn, so laß auch die haupt ader beim daumen.

Ists auf der Ichsel [*Achsel*] oder auf dem Nackhen, so laß die ader baim clainn finger.“

Es werden auch Anweisungen gegeben, „wie man sich in dem Lauff halten soll, mit Essen, damit man sich mit der hilff Gottes vor der Plag bewahren mögt“. Man soll frische, jedoch nicht zu hart gekochte Eier essen, etwas Fleisch von jungen Lämmern und jungen Ziegen, auch sonst gebratenes junges Fleisch, außer vom Schwein, aber auch Wildbret und Fische aus fließenden Gewässern. Alles soll ungesalzen sein, denn „gesalzens ist gifft“. Dazu kann man auch „klaren, nicht zu alten, nicht zu jungen“ Wein trinken, aber nicht zu viel Wasser.

Solche Empfehlungen für die Ernährung zu Pestzeiten gab es bereits acht Jahre vor der Handschrift, nämlich in der Pestordnung von 1521 für Innerösterreich. Es ist jedoch anzunehmen, dass diese den beiden Apothekern in Linz nicht bekannt war. Für das Herzogtum Österreich ob der Enns mit dem Hauptort Linz wie für das Herzogtum Österreich unter der Enns findet man die ersten Angaben für „Speyß vnd Trannckh“ in Pestzeiten erst in der Pestordnung für die Niederösterreichischen Länder von 1540. Die Essensvorschläge beruhen wohl auf eigenen Erfahrungen der Autoren und denen von Kollegen und von Badern und Ärzten.

Das umfängliche Manuskript der beiden Linzer Apotheker mit auch manchen einander widersprechenden Empfehlungen zur Vorbeugung und zur Vermeidung der Pest enthält nicht wie die Pestordnungen die Anordnungen für Maßnahmen lokaler Obrigkeiten, sondern ist wohl für Behandler und vielleicht auch für gebildete Laien bestimmt.

## Footnotes

1 Der am 10.03.1503 in Alcalá de Henares, Spanien, geborene Ferdinand erhielt bei der Habsburgischen Erbteilung die österreichischen Erblande und war somit ab 1521 deren Regent, er wurde 1526 König von Böhmen und Ungarn, 1531 Römischer König und nahm 1556 als Kaiser den Namen Ferdinand I. an. Er starb am 24.07.1564 in Wien.

2 Die „Oberösterreichischen Länder“ waren Tirol und die „zugetanenen und inkorporierten Herrschaften vor dem Arl und Fern“ sowie die österreichischen Vorlande (Letztere sind die Herrschaften in Oberschwaben = Vorderösterreich sowie die Herrschaften im Elsass und im Breisgau).

3 Der „Läß“, das „Zur-Ader-lassen“ kommt von lâzen, lân, lôzen [mhd.] = u. a. (zu)lassen, geschehen lassen, erdulden; als gebräuchliche Kurzform wird „Lassen“ gebraucht (als Substantiv und als Verb); der Aderlasser wird auch Låssl, Lässel genannt.

4 Von louf, lȏf, lœfel [mhd.], Leůff = Lauf, Bewegung, Ereignis; für Seuchen oft als „gemeine“ oder „sterbende“ Läufe auch Sterbleuff, vielfach auch speziell für die Pest gebraucht.

5 Frau Mag. Wilma Buchinger, Informationsservices der Österreichischen Nationalbibliothek, Wien, bin ich, auch hier wie so oft, mit großem Dank verbunden für ihre intensive Recherche in vielen einschlägigen Werken des Fundus der ÖNB, in denen sich keine Hinweise auf die heutigen Namen der beiden Pflanzen finden ließen.

## References

[1] Flamm H. Die ersten Infektions- oder Pest-Ordnungen in den österreichischen Erblanden, im Fürstlichen Erzstift Salzburg und im Innviertel im 16. Jahrhundert. Wien: Verlag d. Österr. Akademie d. Wissenschaften; 2008.

[2] Flamm H. 1529 – der „Englische Schweiß“ im belagerten Wien und bei den osmanischen Belagerern. Wien Med Wochensch. 2020;170:59–70.

[3] Vietz FB. Abbildungen aller medizinisch-ökonomisch-technologischen Gewächse, samt der Beschreibung ihres Nutzens und Gebrauches. Wien: Schrämblischer Verlag; S. 1800–22.

